# Oxidized Amino Acid Residues in the Vicinity of Q_A_ and Pheo_D1_ of the Photosystem II Reaction Center: Putative Generation Sites of Reducing-Side Reactive Oxygen Species

**DOI:** 10.1371/journal.pone.0058042

**Published:** 2013-02-28

**Authors:** Laurie K. Frankel, Larry Sallans, Patrick A. Limbach, Terry M. Bricker

**Affiliations:** 1 Department of Biological Sciences, Division of Biochemistry and Molecular Biology, Louisiana State University, Baton Rouge, Louisiana, United States of America; 2 The Rieveschl Laboratories for Mass Spectrometry, Department of Chemistry, University of Cincinnati, Cincinnati, Ohio, United States of America; University of Hyderabad, India

## Abstract

Under a variety of stress conditions, Photosystem II produces reactive oxygen species on both the reducing and oxidizing sides of the photosystem. A number of different sites including the Mn_4_O_5_Ca cluster, P_680_, Pheo_D1_, Q_A_, Q_B_ and cytochrome b_559_ have been hypothesized to produce reactive oxygen species in the photosystem. In this communication using Fourier-transform ion cyclotron resonance mass spectrometry we have identified several residues on the D1 and D2 proteins from spinach which are oxidatively modified and in close proximity to Q_A_ (D1 residues ^239^F, ^241^Q, ^242^E and the D2 residues ^238^P, ^239^T, ^242^E and ^247^M) and Pheo_D1_ (D1 residues ^130^E, ^133^L and ^135^F). These residues may be associated with reactive oxygen species exit pathways located on the reducing side of the photosystem, and their modification may indicate that both Q_A_ and Pheo_D1_ are sources of reactive oxygen species on the reducing side of Photosystem II.

## Introduction

In higher plants and cyanobacteria the Photosystem II (PS II) complex contains more than twenty polypeptide subunits. At the core of the photosystem, six intrinsic membrane proteins are unequivicolly required for oxygen evolution: the D1 and D2 proteins, the CP43 and CP47 proteins and the α- and β- subunits of cytochrome b_559_. The genetic deletion of these components results in loss of the assembly of the photosystem *in vivo*, while their biochemical removal from isolated PS II complexes results in the loss of PS II function *in vitro* [1], [2]. Over the past twelve years, crystal structures of cyanobacterial PS II have enhanced our understanding of the molecular organization of the polypeptides of the photosystem and the active sites for oxygen evolution (the Mn_4_O_5_Ca cluster) and for quinone reduction [3], [4], [5], [6], [7]. Recently, a high resolution 1.9 Å crystal structure of cyanobacterial PS II has been presented [8]. Crystal structures for higher plant PS II, however, are not available. There are significant differences between the extrinsic protein complement of the higher plant and the cyanobacterial photosystems [9]. While both contain the PsbO protein [10], the PsbU and PsbV proteins are present in cyanobacteria while the PsbP and PsbQ proteins are present in higher plants. Cyanobacterial versions of these latter two proteins (CyanoP and CyanoQ) are present, but their functional roles in cyanobacterial PS II is unclear [9]. PsbP and PsbQ in higher plants and PsbU and PsbV in the cyanobacteria appear to facilitate the accumulation of essential inorganic cofactors for oxygen evolution (Ca^+2^ and Cl^−^) [9], [11] and may also perform other functions within the photosystem [12], [13]. With respect to the major intrinsic components, however, the cyanobacterial and higher plant systems are quite similar. The amino acid sequences of the intrinsic components (D1, D2, CP43 and CP47) are nearly identical in both groups (>85% similarity, [14]). Consequently, one would expect that these core structural elements of PS II would be highly homologous between higher plants and cyanobacteria.

PS II is the major site of photoinhibition in all oxygenic organisms and appears particularly susceptible to damage by reactive oxygen species (ROS). The production of molecular oxygen by PS II is accompanied by the unavoidable possibility of oxidative modification of amino acid residues within the PS II complex in the vicinity of the Mn_4_O_5_Ca cluster, the oxygen-evolving site of the photosystem [15], [16]. Singlet oxygen (^1^O_2_) produced at P_680_, the primary electron donor of the photosystem, has also been proposed as a source of ROS produced by the photosystem [17], [18], [19]. Recently, we identified a number of oxidized CP43 residues (^354^E, ^355^T, ^356^M and ^357^R) which are located in close proximity to the manganese cluster and which may be associated with an oxygen/ROS egress channel on the oxidizing side of the photosystem [20]. Barry and coworkers have also identified oxidatively modified tryptophan residues on both the CP43 (^365^W) and D1 (^317^W) proteins which appear to be targets for oxidizing side ROS [21], [22], [23]. Additionally, reductants produced by PS II, such as Q_B_^−2^ [24], Pheo_D1_^−^ [25], Q_A_^−^ [26], and, possibly, reduced low potential cytochrome b_559_ [27], [28], appear to have redox potentials and lifetimes sufficient to reduce molecular oxygen and have been hypothesized to be sources of ROS. Sharma et al. [29] had previously identified a D1 peptide (^130^E–^136^R) which lies in the vicinity of PheoD1 and which contained a single oxidative modification on an unidentified residue.

One would predict that amino acid residues in the vicinity of the sites of ROS production should be particularly susceptible to ROS modification. The identification of such oxidatively modified residues in the photosystem should serve to identify both the putative site(s) of ROS generation and possibly the putative path(s) for ROS exit from PS II. In this communication we report the presence of natively oxidized amino acids in the vicinity of both Q_A_ and Pheo_D1_ in PS II membranes isolated from field-grown spinach. These modifications are apparently normally present in the PS II isolated from field-grown material and our findings suggest that Q_A_ and Pheo_D1_ may be sources of ROS under native growth conditions.

## Results

**Figure 1:**
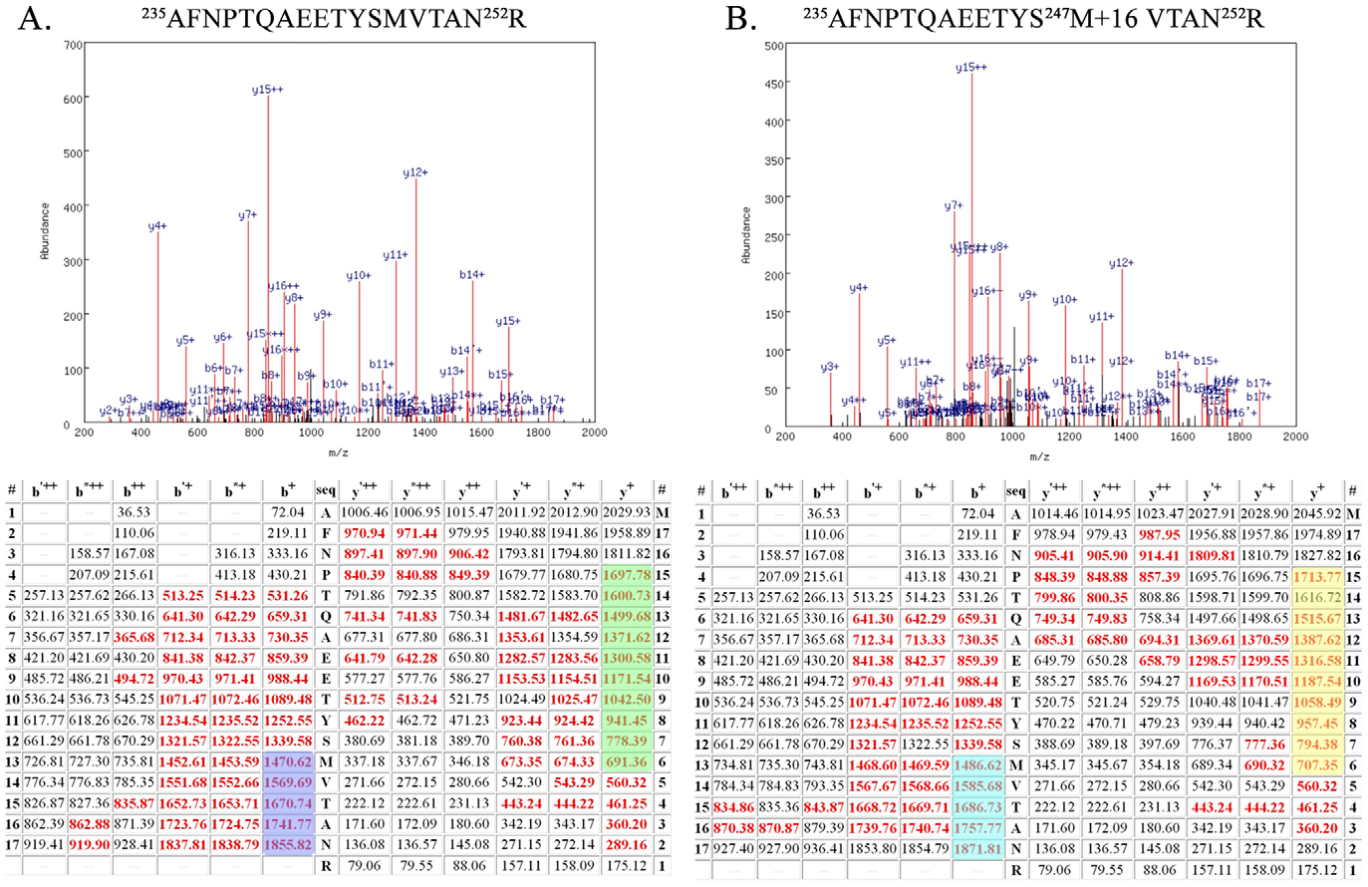
Example Mass Spectrometry Data from the Unmodified Peptide. ^235^AFNPTQAEETYSMVTAN^252^R and the Oxidatively Modified Peptide ^235^AFNPTQAEETYS^247^M+16 VTAN^252^R of the D2 Protein A. Top, spectrum of the collision-induced dissociation (CID) of the unmodified peptide ^235^AFNPTQAEETYSMVTAN^252^R. Various identified ions are labeled. Bottom, table of all predicted masses for the y- and b- ions generated from this peptide sequence. Ions identified in the CID spectrum (above) are shown in red. The b'^++^, b'^+^ y'^++^ and y'^+^ ions are generated by the neutral loss of water while the b*^++^, b*^+^ y*^++^ and y*^+^ ions are generated from the loss of ammonia. B. Top, spectrum of the CID dissociation of the modified ^235^AFNPTQAEETYS^247^M+16 VTAN^252^R. Various identified ions are labeled. Bottom, table of all predicted masses for the y- and b- ions generated from this peptide sequence. Ions identified in the CID spectrum are shown in red. The b'^++^, b'^+^ y'^++^ and y'^+^ ions are generated by the neutral loss of water while the b*^++^, b*^+^ y*^++^ and y*^+^ ions are generated from the loss of ammonia. For comparison the b^13+^–b^17+^ ions of the unmodified peptide are highlighted in blue and those of the modified peptide are highlighted in cyan. All b ions longer than b^12+^ in the modified peptide are 16 Da larger than the corresponding ions observed from the unmodified peptide. This indicates that ^247^M contains an oxidative modification. Additionally, the y^6+^–y^15+^ions of the unmodified peptide are highlighted in green, while those of the modified peptide are highlighted in yellow. All y ions longer than y^5+^ in the modified peptide are 16 Da larger than the corresponding ions observed from the unmodified peptide. This verifies that ^247^M contains an oxidative modification. The p values for the unmodified and modified peptide were 10^−13^ and 10^−11^, respectively.

Fig. 1 illustrates the quality of the mass spectrometry data used for the identification of oxidized amino acid residues in the D1 and D2 proteins. In this figure the MS/MS data collected for the D2 peptide ^235^A–^252^R are illustrated. In Fig. 1A, the data from the unmodified peptide are illustrated, while in Fig. 1B, data from the peptide bearing oxidized ^247^M are shown. In this example and others (Figs. S1–S2), both modified and unmodified versions of the target peptide were identified; in another example only the modified peptide was detected (Fig. S3). Using a p value ≤0.00001 assured extremely high quality peptide identifications with nearly complete y- and b-ion series being observed.

In this communication, we have focused on the domains of the D1 and D2 proteins located at or near redox active cofactors located on the stromal face of the thylakoid membrane. A total of 10 oxidatively modified amino acid residues on the D1 and D2 proteins were observed in the vicinity of these cofactors. These are summarized in Table 1 along with the type of oxidative modifications and the residue location (surface or buried and not in contact with cavity or channel) within the *T. vulcanus* crystal structure. Please note that these residues were originally reported to be modified in Frankel et al. [20], however, their close association with the reducing-side cofactors was not discussed in that communication.

**Table 1:** Oxidatively Modified Residues in the Vicinity of Pheo_D1_ and Q_A_.

Protein	Modified Spinach Residues	Corresponding *Thermosynechococcus* Residues
**D1**	^130^E^a^ + go	^130^Q
	^133^L^a^ + go	^133^L
	^135^F^a^ + go	^135^Y
	^239^F^b^ + go	^239^F
	^241^Q^b^ + ca	^241^Q
	^242^E^b^ + gam	^242^E
**D2**	^238^P^b^ + ca	^237^P
	^239^T^b^ + go	^238^T
	^242^E^b^ + gam	^241^E
	^247^M^b^ + go	^246^M

Individual residues are listed along with the modifications observed. For a complete list of oxidative modification types, the amino acids targeted, and mass modifications searched for in this study, as well as structures arising from these oxidative modifications, see [50], [51]. Key: ca, carbonyl addition (+14 Da); gam, Glu/Asp modification (decarboxylation and oxidation, −30 Da); go, general oxidation (+16 Da). Oxidative modification of these residues was originally reported in Frankel et al. [20].

a Buried residues not adjacent to apparent cavities/channels.

b Surface-exposed residues.

In general, mass spectrometry coverage of intrinsic membrane proteins is quite challenging. The overall sequence coverage observed in this study for the proteins examined was 24% for D1 and 27% for D2, values which are quite comparable to that observed for these proteins by other investigators (see, for instance Nakamura et al. [30]). However, the coverage of the residues located in the stromally exposed domains of these proteins was significantly higher, 67% for D1 and 48% for D2. These are the domains of principal interest in this study, as ROS produced on the reducing side of the photosystem must transit these regions to exit the photosystem. Within this context it should be noted that most oxidative modifications to amino acid residues lying in the transmembrane helixes of these proteins would be difficult to identify and many would escape detection due to their high hydophobicity and consequent expected poor resolution during reversed-phase chromatography.

**Figure 2:**
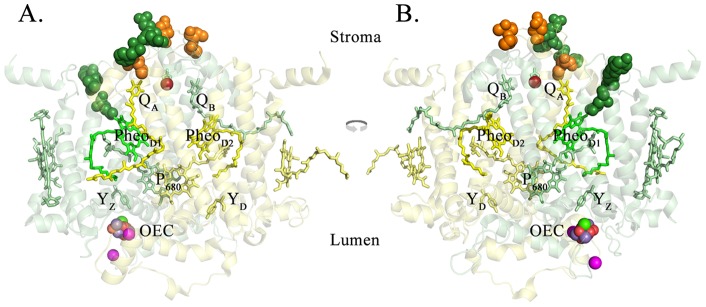
Oxidized Residues Identified on the Stromally Exposed Regions of the D1 and D2 Proteins in the Vicinity of Q_A_ and Pheo_D1_. The *T. vulcanus* residues corresponding to the oxidatively modified spinach residues (Table 1) are highlighted. These oxidized residues are shown as spheres superimposed on monomer I of the *T. vulcanus* structure. For clarity, only the D1 and D2 proteins and their associated cofactors are shown. A. the view from outside Monomer I, looking towards the dimeric complex from within the plane of the membrane. B. the view from Monomer II looking towards its interface with Monomer I within the plane of the membrane. The D1 protein is shown in pale green and the D2 protein is shown in pale yellow. The oxidatively modified residues of D1 are shown in dark green while those of D2 are shown in orange. Various cofactors of both D1 and D2 are labeled and colored pale green or yellow, respectively. Pheo_D1_ is shown in bright green. The non-heme iron is shown in bright red. The Mn_4_O_5_Ca cluster and its associated chloride ions are labeled as the OEC. Figs. 2–4 were produced using PYMOL [53].

The D1 and D2 proteins are highly homologous between higher plants and cyanobacteria (>95% similarity). Consequently, we can directly map the modified residues observed to be modified in spinach onto the homologous residues in the *T. vulcanus* crystal structure. These results are shown in Fig. 2. The close proximity of oxidatively modified residues to Q_A_ and Pheo_D1_ is evident in this illustration. Additionally, it is apparent that these residues appear to form two rather distinct groups. The first group appears associated with Q_A_, consists of both D1 and D2 residues, and leads in a nearly continuous manner from the cofactor to the surface of the complex. The second group is associated with Pheo_D1_, consists solely of D1 residues, also forming a near continuous grouping of modified residues. This group of residues, however, does not reach the surface of the PS II complex.

## Discussion

It must be stressed that this is an observational study of the oxidative modifications naturally occurring in PS II isolated from field grown-spinach. We cannot comment on the field conditions which lead to these oxidative modifications (high light intensity, high temperatures, nutrient limitations, presence of heavy metals, etc.) nor on the chemical identification of the ROS responsible. Studies addressing some of these questions will be the topic of additional communications.

The oxidative modification of these D1 and D2 residues indicates that they have been modified by ROS. The proximity of these residues to Q_A_ (D1 residues: ^239^F, ^241^Q, ^242^E and the D2 residues: ^238^P,^ 239^T,^ 242^E, and^ 247^M) and Pheo_D1_ (D1 residues:^130^E, ^133^L and ^135^F) supports the hypothesis that these two cofactors are sites of ROS production on the reducing side of PS II.

**Figure 3:**
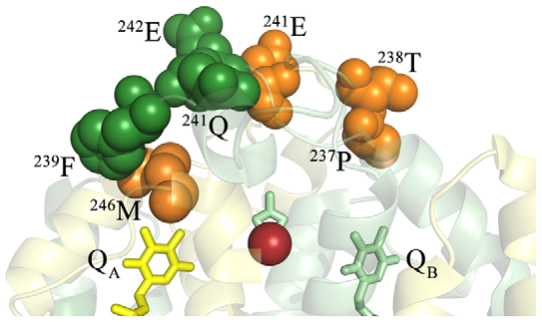
Detail of the Oxidized Residues in the Vicinity of Q_A_. A close-up of the Q_A_ – Non-Heme Iron – Q_B_ region is shown. The *T. vulcanus* residues corresponding to the oxidatively modified spinach residues (Table 1) are highlighted and labeled. The D1 protein is shown in pale green and the D2 protein is shown in pale yellow. The oxidatively modified residues of D1 are shown in dark green while those of D2 are shown in orange, with the individual modified residues being labeled. Q_A_ is shown in yellow, Q_B_ in green and the non-heme iron is shown in bright red.

With respect to the residues in the vicinity of Q_A_ in *Thermosynechococcus*, it should be noted that there is a one amino acid deletion at residue 10 in the D2 sequence with respect to the spinach sequence (Table 1). Consequently, in *Thermosynechococcus*: Q_A_ – 2.9Å – D2:^246^M – D1:^239^F – D1:^241^Q – D1:^242^E – D2:^241^E. These residues appear to form a near contiguous chain of oxidized residues leading from Q_A_ (Fig. 3). Two additional oxidized residues D2:^237^P and ^238^T may also be part of this oxidized residue complex but these are more distantly located. The mass spectra identifying these modified residues are shown in Figs. 1, S1 and S2. All of these residues are at least partially surface-exposed. D2:^246^M, however, exhibits very limited contact with the bulk solvent. It should be noted that the oxidized D2 residues ^237^P and ^238^T are also in relatively close proximity to Q_B_ (9 Å and 13 Å, respectively, Fig. 3). While no additional oxidized residues in the immediate vicinity of Q_B_ were observed leading to the surface-located residues D2:^237^P and ^238^T, it is formally possible that oxidized residues are present in the region near Q_B_, but that they escaped detection in our experiments.

**Figure 4:**
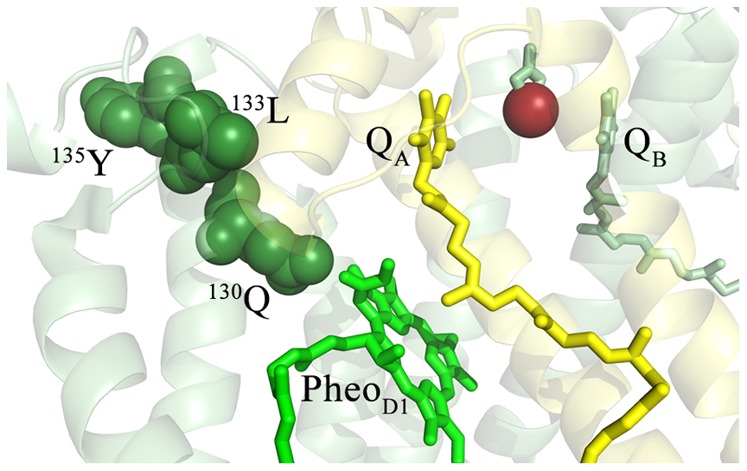
Detail of the Oxidized Residues in the Vicinity of Pheo_D1_. The *T. vulcanus* residues corresponding to the oxidatively modified spinach residues (Table 1) are highlighted and labeled. The D1 protein is shown in pale green and the D2 protein is shown in pale yellow. The oxidatively modified residues of D1 are shown in dark green, with the individual modified residues being labeled. Pheo_D1_ is shown in bright green, Q_A_ is shown in yellow, Q_B_ in green and the non-heme iron is shown in bright red. For clarity, modified residues in the vicinity of Q_A_ (and detailed in Fig. 3) are not shown.

With respect to the residues in close proximity to Pheo_D1_, D1:^130^E in spinach and *Chlamydomnas* is replaced by D1:^130^Q in the *T. vucanus* structure. It should be noted that in *T. vulcanus*, D1:^130^Q is present in the constitutively expressed D1–1 isoform while the D1–2 and D1–3 isoforms, which are expressed only under certain environmental conditions, contain D1:^130^E [31], [32]. These residues have been reported to be hydrogen- bonded to Pheo_D1_ [33], [34]. Additionally, in *Thermosynechococcus*, D1:^135^Y is replaced by D1:^135^F (Table 1). Consequently, in the *Thermosynechococcus* structure: Pheo_D1_ – 2.9Å – D1:^130^Q – D1:^133^L –D1:^135^F (Fig. 4). The mass spectrum identifying this group of modified residues is shown in Fig. S3. These oxidized residues may be adjacent to a putative ROS exit pathway leading from Pheo_D1_ to residues near or at the surface of the complex. It should be noted that in the static crystal structure, none of these residues are surface-exposed nor are they in contact with any apparent cavities or channels. Other residues which were not detected in our studies may be associated with the putative pathway, completing a pathway to the surface of the complex.

It is unclear at this time how molecular oxygen penetrates into the protein structure to reach the vicinity of Pheo_D1_. While no channels or cavities are present in the static protein structure in the vicinity of Pheo_D1_, it is possible that these form transiently either due to thermal motion of the PS II complex on a nsec timescale or as a result of conformational changes occurring during the S-state transitions. It is also possible that oxygen can diffuse directly into the protein matrix as has been demonstrated in other systems [35]. In any event, our observation that the D1 residues ^130^E, ^133^L and ^135^F are oxidatively modified strongly suggests that molecular oxygen can penetrate the PS II structure and become partially reduced to an ROS by Pheo_D1_.

Earlier studies identified domains containing oxidatively modified D1 and D2 residues. Sharma et al. [36] determined that the D1 peptide ^130^E–^136^R contained an oxidative modification, however the actual residue(s) modified and its spatial relationship with Pheo_D1_ were not determined. Our observation that oxidative modification occurs on the D1 residues ^130^E, ^133^L and ^135^F fully confirms this observation of Sharma et al. [36]. These authors also identified a number of other peptides containing putative oxidative modifications on both the D1 and D2 proteins. However, none of the other residues that we observe to be oxidatively modified on the stromal domain lie in these additional peptides.

PS II, particularly when under stress, apparently can produce a variety of ROS at a variety of sites [16]. Several studies have identified the production of ROS, particularly the short-lived OH^•^, by the donor side of PS II [37], [38]. ^1^O_2_ produced by the reaction of molecular oxygen with ^3^P_680_ has also been observed [17], [18], [19]. Interestingly, no oxidative modifications in the vicinity of P_680_ have been observed [20]. Since the production of ^1^O_2_ by PS II is well established [39], the lack of observed modifications in the vicinity of P_680_ could be a detection issue, as the peptides in the vicinity of P_680_ are highly hydrophobic and difficult to resolve by reversed-phase HPLC. It is also possible that ^1^O_2_ may be vectored away from P_680_ rapidly, giving rise to a low yield of oxidative modifications. Finally, oxidative modification of residues in the vicinity of P_680_ may trigger D1 turnover more effectively than oxidative modifications at other sites *in vivo*. We cannot distinguish between these and other possibilities at this time.

ROS may also be produced on the reducing side of the photosystem by the partial reduction of molecular oxygen, yielding long-lived O_2_^•−^ and H_2_O_2_ and the very short-lived OH^•^. It should be stressed, however, that at this time we cannot discriminate between these or other mechanisms that produce the ROS responsible for the oxidative modifications that we observe. Using mass spectrometry, it is also very difficult, and in most cases impossible, to differentiate oxidative modifications of amino acid side chains produced by OH^•^, ^1^O_2_, O_2_^•−^ or other oxidative species [40], [41].

The site of ROS production by PS II has been the subject of much discussion [16]. Earlier, we reported that CP43:^ 354^E, ^355^T, ^356^M and ^357^R, which are in close proximity to the Mn_4_O_5_Ca cluster, were oxidatively modified [20]. These results indicate that the manganese cluster itself appears to be a source of oxidizing-side ROS. On the reducing side of the photosystem, Pheo_D1_, Q_A_, Q_B_^−2^ and low potential cytochrome b_559_ have all been suggested as sites of ROS production. In this communication we have reported the oxidative modification of residues in close proximity to Pheo_D1_ and Q_A_. These results support the hypothesis that both of these sites can produce ROS that lead to amino acid residue oxidative modification. Since these modifications were observed on PS II membranes isolated from market spinach, it appears that they can accumulate to detectable levels within the D1 protein lifetime (t_1/2_≈2 hr [42]). Interestingly, no oxidative modifications in the vicinity of the Mn_4_O_5_Ca cluster were observed on the D1 protein on this same plant material. Again, it is possible that D1 modifications in the vicinity of the metal cluster (or, perhaps, P_680_) may trigger D1 turnover and, consequently, limit the detection and/or accumulation of such putative oxidative modifications.

While no modified residues were observed in the immediate vicinity of Q_B_, we cannot rule out, at this time, the possibility that this site could also contribute to reducing-side ROS production. Additionally, since we did not collect mass spectrometry data on the cytochrome b_559_ α and β subunits or on the other low molecular mass subunits in the vicinity of this cytochrome, we cannot comment on their ability to produce ROS. We also cannot speculate on the relative rate of ROS production by Pheo_D1_ or Q_A_ (or other putative ROS-producing sites). We have no quantitative data as to the proportion of modified amino acid residues present at any of the observed positions. Indeed, such quantification would be difficult to obtain given the different hydophobicity of the unmodified *vs.* modified peptides and their consequent differential resolution by reversed-phase chromatography. Elucidation of the time-course for modification of these oxidized residues using ^18^O_2_, however, may provide valuable evidence bearing on the relative importance of ROS production by Pheo_D1_ and Q_A_. These experiments are currently underway.

## Materials and Methods

PS II membranes were isolated from market spinach [43], [44]. The PS II membranes were suspended at 2 mg chlorophyll/ml in 50 mM Mes-NaOH, pH 6.0, 300 mM sucrose, 15 mM NaCl buffer and frozen at −80°C until use. The proteins in the samples were separated on a 12.5–20% polyacrylamide gradient by lithium dodecyl sulfate-polyacrylamide gel electrophoresis [45] with the modifications outlined by Rabilloud et al. [46] and Sun et al. [47]. Electrophoresis was performed for 16 hrs at 1 W at 4°C. Upon completion of electrophoresis, the gels were stained with Coomassie Blue, destained, and protein bands containing D1 and D2 proteins were excised. These proteins (along with a number of other protein components of PS II) were then processed *en masse* for trypsin digestion using standard methods. In some cases, the tryptic peptides were processed using a C18 ZipTip® prior to mass analysis.

Chromatography was performed as previously described [20]. Briefly, the tryptic peptides were resolved on a Waters reversed phase, X-Bridge C18 column. The mobile phases consisted of a 95:5 water:acetonitrile with 0.1% formic acid aqueous phase and a 95:5 acetonitrile:water with 0.1% formic acid organic phase, and the peptides were eluted with the gradient described previously [20]. Mass spectrometry was performed on a Thermo Scientific LTQ-FT™, a hybrid instrument consisting of a linear ion trap and a Fourier transform ion cyclotron resonance mass spectrometer. Instrumental conditions and data collection were described in Frankel et. al. [20].

We performed two biological replicates. The MassMatrix Program ver. 1.3.1 [48], [49] was used in the identification and analysis of the resultant peptides. The program was modified to search for the oxidative modifications which have been reported in the literature [50], [51]. A FASTA library containing the spinach PS II protein sequences was searched, as was a decoy library. The decoy library contained these same PS II components but with their amino acid sequences reversed. No hits to the decoy library were observed. Such decoy libraries are used to evaluate the frequency of false positives expected when searching the real FASTA database [52]. For the determination of the quality of the peptide calls within MassMatrix, max(pp_1_, pp_2_) was ≥8.5 and pp_Tag_ ≥5.0 [48], [49]. These parameters yield a p value of ≤0.00001; only oxidized peptides which exhibited this extremely low p value were considered. Since the data was of very high quality, the union of the replicate data sets was examined [20]. Since the D1 and D2 proteins present in spinach and *T. vulcanus* are highly homologous, the PYMOL software suite [53] was used to map the oxidatively modified residues observed in spinach onto the *T. vulcanus* PS II structure [8] of the D1 and D2 proteins.

It should be noted that protein electrophoresis is the principal source of protein oxidation artifacts in many biochemical studies. The ammonium persulfate catalyst (and the TEMED activator) typically used in this method for the polymerization of the acrylamide-bis acrylamide monomers generates sulfate radicals. This radical can react with water to produce both O_2_^•−^ and OH^•^ [54], both of which can oxidatively modify proteins. To alleviate this problem the polyacrylamide gels used in this study were thoroughly degassed and photopolymerized with flavin mononucleotide, diphenyliodonium chloride and sodium toluenesulfinic acid [46]. Additionally, the cathode buffer contained thioglycolate [47]. This electrophoretic system had been shown to completly eliminate artifactual electrophoresis-associated oxidative modifications of cytochrome c [47] and greatly minimize apparent electrophoresis-induced oxidative modifications in PS II (see [20] Fig. S1). Subsequent to electrophoresis, the protein and peptide samples were maintained under reducing conditions (presence of dithiothreitol and/or low pH) to minimize artifactual oxidative modifications. Staining was performed in the presence of acetic acid, the excised protein bands were reduced with dithiothreitol (and then blocked with iodoacetic acid), and after tryptic digestion the peptides were brought to 0.1% formic acid and frozen at −80°C. Reversed phase HPLC was performed in the presence of 0.1% formic acid. The sheath and auxiliary gas for electrospray ionization was N_2_ [20].

## Supporting Information

Figure S1 **Mass Spectrometry Data from the Unmodified Peptide.** ^235^AFNPTQAEETYSMVTAN^252^R and the Oxidatively Modified Peptide ^235^AFN^238^P+16 ^239^T+16 QA^242^E-30 ETYSM+16 VTAN^252^R of the D2 Protein A. Top, spectrum of the CID dissociation of the unmodified peptide ^235^AFNPTQAEETYSMVTAN^252^R. Various identified ions are labeled. Bottom, table of all predicted masses for the y- and b- ions generated from this peptide sequence. Ions identified in the CID spectrum (above) are shown in red. The b'^++^, b'^+^ y'^++^ and y'^+^ ions are generated by the neutral loss of water while the b*^++^, b*^+^ y*^++^ and y*^+^ ions are generated from the loss of ammonia. B. Top, spectrum of the CID dissociation of the modified ^235^AFN^238^P+16 ^239^T+16 QA^242^E-30 ETYSM+16 VTAN^252^R. Various identified ions are labeled. Bottom, table of all predicted masses for the y- and b- ions generated from this peptide sequence. Ions identified in the CID spectrum are shown in red. The b'^++^, b'^+^ y'^++^ and y'^+^ ions are generated by the neutral loss of water while the b*^++^, b*^+^ y*^++^ and y*^+^ ions are generated from the loss of ammonia. The p values for the unmodified and modified peptide were 10^−13^ and 10^−14^, respectively.
(DOCX)

Figure S2 **Mass Spectrometry Data from the Unmodified Peptide.** ^239^FGQEEETYNIHAAHGYFG^257^R and the Oxidatively Modified Peptide ^239^F+16 G^241^Q+14 ^242^E-30 EETYNIHAAHGYFG^257^R of the D1 Protein A. Top, spectrum of the CID dissociation of the unmodified peptide ^239^FGQEEETYNIHAAHGYFG^257^R. Various identified ions are labeled. Bottom, table of all predicted masses for the y- and b- ions generated from this peptide sequence. Ions identified in the CID spectrum (above) are shown in red. The b'^++^, b'^+^ y'^++^ and y'^+^ ions are generated by the neutral loss of water while the b*^++^, b*^+^ y*^++^ and y*^+^ ions are generated from the loss of ammonia. B. Top, spectrum of the CID dissociation of the modified G^241^Q+14 ^242^E-30 EETYNIHAAHGYFG^257^R. Various identified ions are labeled. Bottom, table of all predicted masses for the y- and b- ions generated from this peptide sequence. Ions identified in the CID spectrum are shown in red. The b'^++^, b'^+^ y'^++^ and y'^+^ ions are generated by the neutral loss of water while the b*^++^, b*^+^ y*^++^ and y*^+^ ions are generated from the loss of ammonia. The p values for the unmodified and modified peptide were 10^−8^ and 10^−9^, respectively.
(DOCX)

Figure S3 **Mass Spectrometry Data from the Oxidatively Modified Peptide ^130^E+16 WE^133^L+16 S^135^F+16 ^136^R of the D1 Protein A.** Top, spectrum of the CID dissociation of the modified peptide. Various identified ions are labeled. Bottom, table of all predicted masses for the y- and b- ions generated from this peptide sequence. Ions identified in the CID spectrum (above) are shown in red. The b'^++^, b'^+^ y'^++^ and y'^+^ ions are generated by the neutral loss of water while the b*^++^, b*^+^ y*^++^ and y*^+^ ions are generated from the loss of ammonia. The p value for this peptide is 10^−6^.
(DOCX)
