# Supplementary figures and images for: Oxidized Amino Acid Residues in the Vicinity of QA and PheoD1 of the Photosystem II Reaction Center: Putative Generation Sites of Reducing-Side Reactive Oxygen Species
Source: PLoS One. 2013 Feb 28;8(2):e58042. doi: 10.1371/journal.pone.0058042 (PMC3585169; doi:10.1371/journal.pone.0058042)

Figure S1.


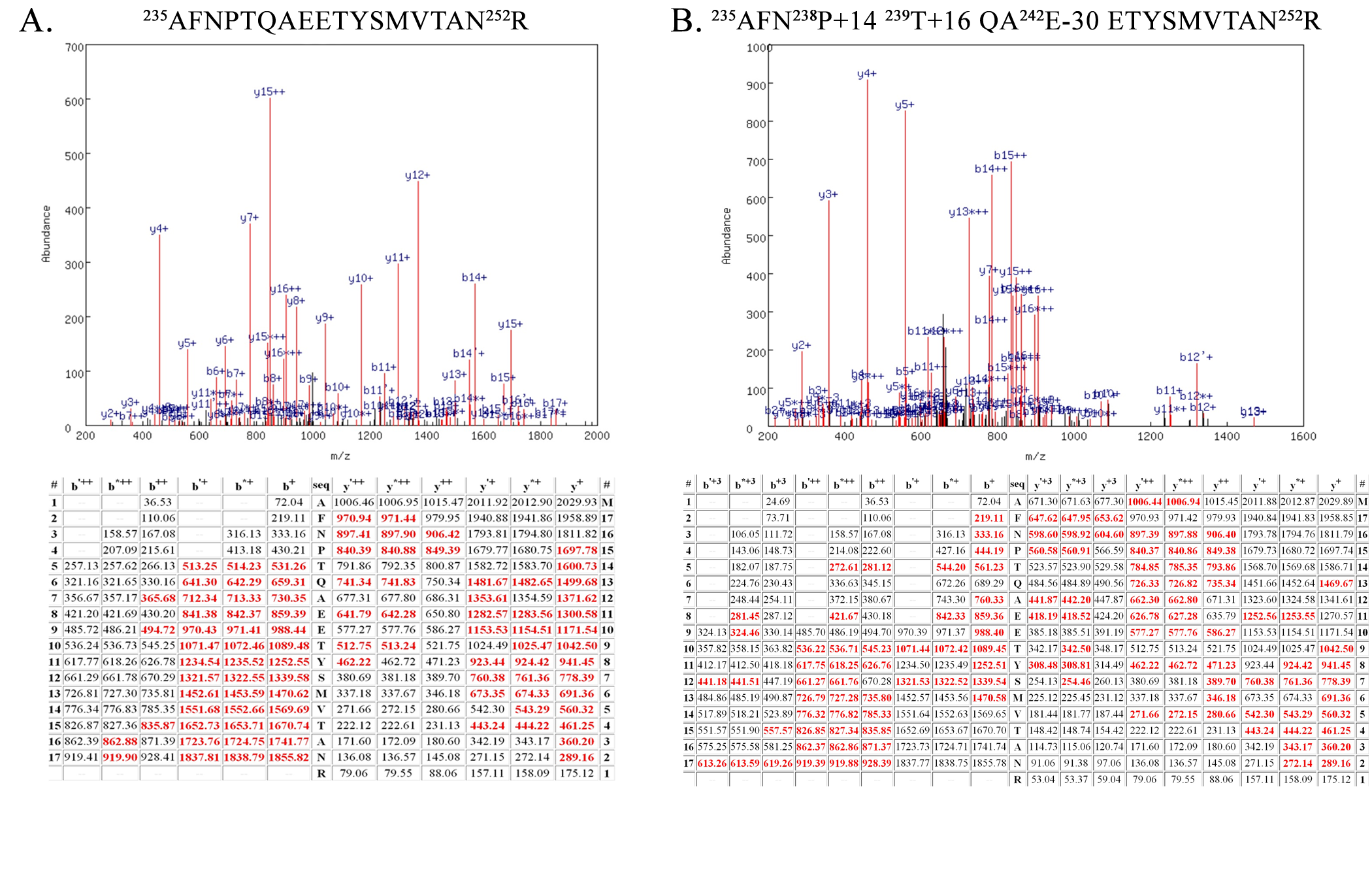

Supplement: Figure S1 — Mass Spectrometry Data from the Unmodified Peptide. 235AFNPTQAEETYSMVTAN252R and the Oxidatively Modified Peptide 235AFN238P+16 239T+16 QA242E-30 ETYSM+16 VTAN252R of the D2 Protein A. Top, spectrum of the CID dissociation of the unmodified peptide 235AFNPTQAEETYSMVTAN252R. Various identified ions are labeled. Bottom, table of all predicted masses for the y- and b- ions generated from this peptide sequence. Ions identified in the CID spectrum (above) are shown in red. The b'++, b'+ y'++ and y'+ ions are generated by the neutral loss of water while the b*++, b*+ y*++ and y*+ ions are generated from the loss of ammonia. B. Top, spectrum of the CID dissociation of the modified 235AFN238P+16 239T+16 QA242E-30 ETYSM+16 VTAN252R. Various identified ions are labeled. Bottom, table of all predicted masses for the y- and b- ions generated from this peptide sequence. Ions identified in the CID spectrum are shown in red. The b'++, b'+ y'++ and y'+ ions are generated by the neutral loss of water while the b*++, b*+ y*++ and y*+ ions are generated from the loss of ammonia. The p values for the unmodified and modified peptide were 10−13 and 10−14, respectively. (DOCX) [file pone.0058042.s001.docx]

Figure S2.


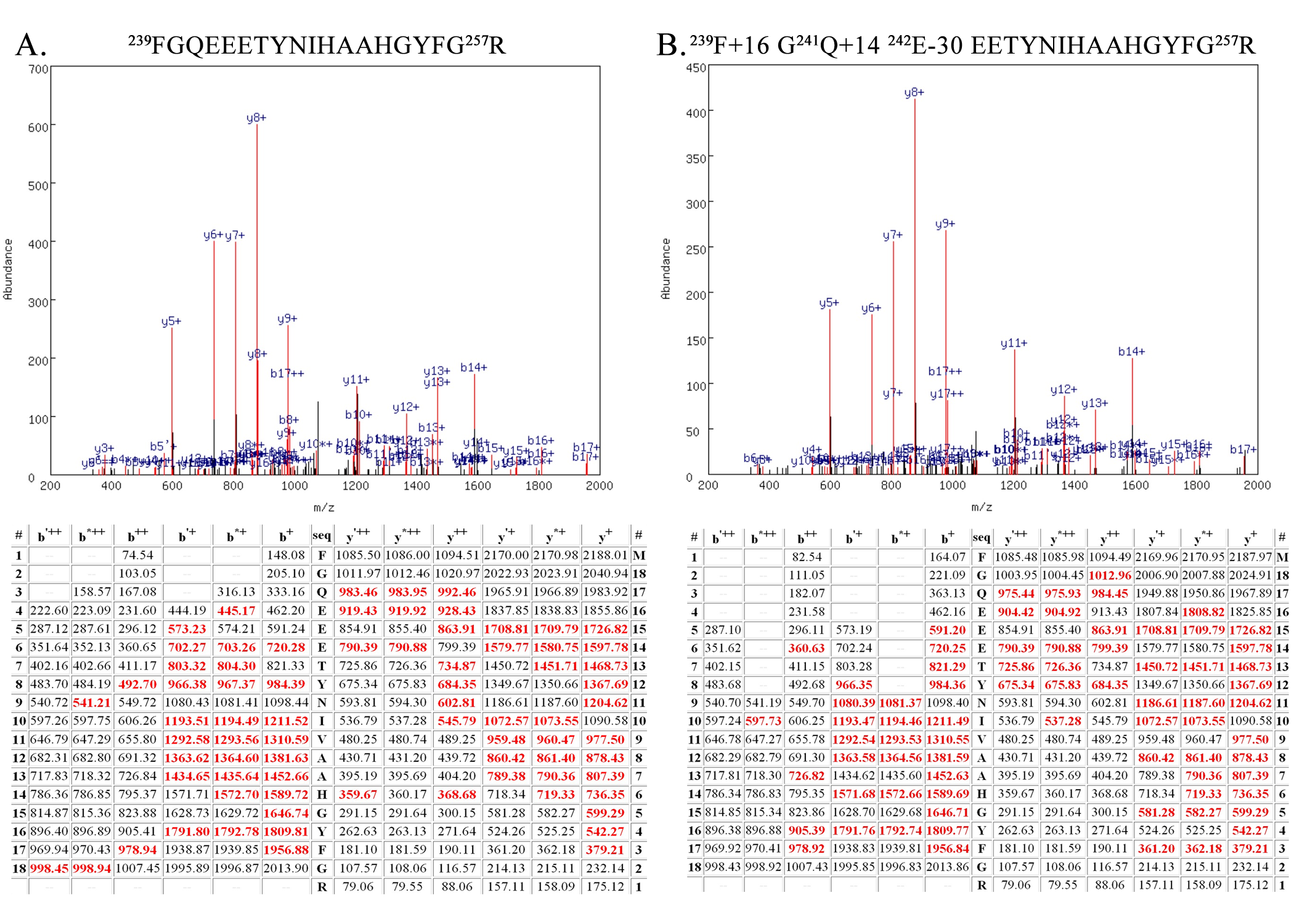

Supplement: Figure S2 — Mass Spectrometry Data from the Unmodified Peptide. 239FGQEEETYNIHAAHGYFG257R and the Oxidatively Modified Peptide 239F+16 G241Q+14 242E-30 EETYNIHAAHGYFG257R of the D1 Protein A. Top, spectrum of the CID dissociation of the unmodified peptide 239FGQEEETYNIHAAHGYFG257R. Various identified ions are labeled. Bottom, table of all predicted masses for the y- and b- ions generated from this peptide sequence. Ions identified in the CID spectrum (above) are shown in red. The b'++, b'+ y'++ and y'+ ions are generated by the neutral loss of water while the b*++, b*+ y*++ and y*+ ions are generated from the loss of ammonia. B. Top, spectrum of the CID dissociation of the modified G241Q+14 242E-30 EETYNIHAAHGYFG257R. Various identified ions are labeled. Bottom, table of all predicted masses for the y- and b- ions generated from this peptide sequence. Ions identified in the CID spectrum are shown in red. The b'++, b'+ y'++ and y'+ ions are generated by the neutral loss of water while the b*++, b*+ y*++ and y*+ ions are generated from the loss of ammonia. The p values for the unmodified and modified peptide were 10−8 and 10−9, respectively. (DOCX) [file pone.0058042.s002.docx]

Figure S3.


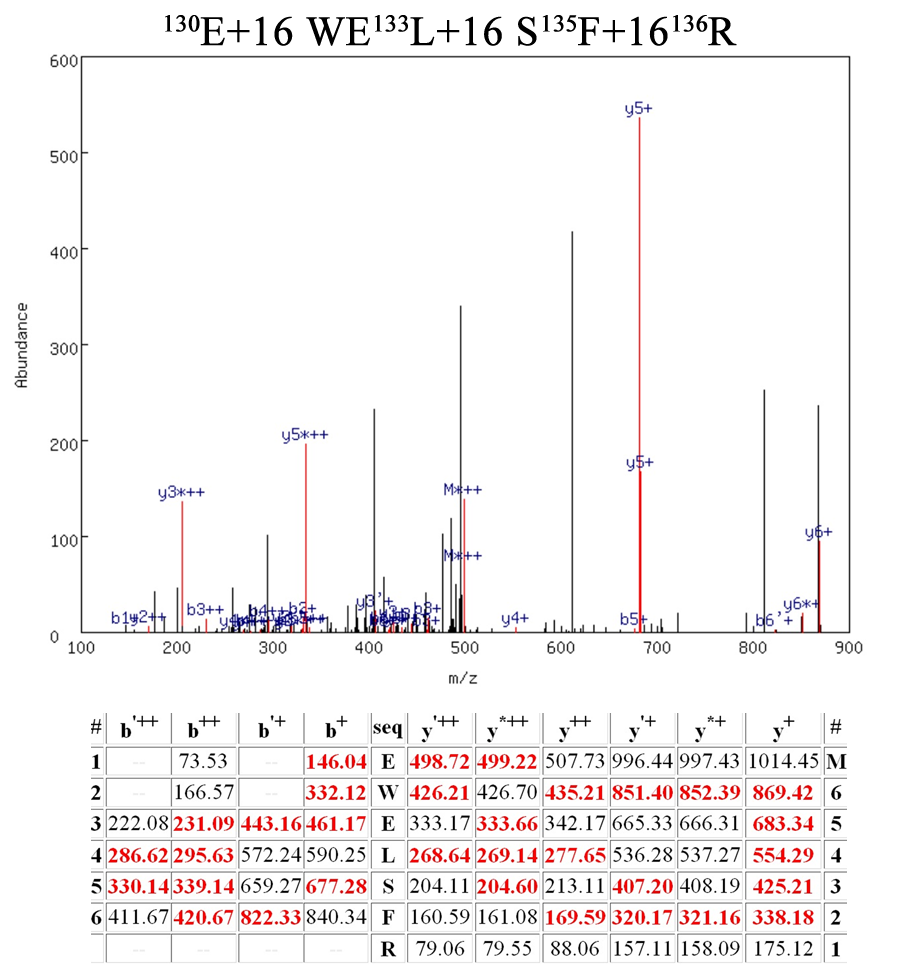

Supplement: Figure S3 — Mass Spectrometry Data from the Oxidatively Modified Peptide 130E+16 WE133L+16 S135F+16 136R of the D1 Protein A. Top, spectrum of the CID dissociation of the modified peptide. Various identified ions are labeled. Bottom, table of all predicted masses for the y- and b- ions generated from this peptide sequence. Ions identified in the CID spectrum (above) are shown in red. The b'++, b'+ y'++ and y'+ ions are generated by the neutral loss of water while the b*++, b*+ y*++ and y*+ ions are generated from the loss of ammonia. The p value for this peptide is 10−6. (DOCX) [file pone.0058042.s003.docx]
